# A Randomized Clinical Trial Preventive Outreach Targeting Dental Caries and Oral-Health-Related Quality of Life for Refugee Children

**DOI:** 10.3390/ijerph18041686

**Published:** 2021-02-10

**Authors:** Murad Alrashdi, Maria Jose Cervantes Mendez, Moshtagh R. Farokhi

**Affiliations:** 1 Department of Orthodontic and Paediatric Dentistry, College of Dentistry, Qassim University, KSA, Qassim 51452, Saudi Arabia; 2 Department of Developmental Dentistry, School of Dentistry at the University of Texas Health Science Center at San Antonio, San Antonio, TX 78229, USA; CervantesMen@uthscsa.edu; 3 Department of Comprehensive Dentistry, School of Dentistry at the University of Texas Health Science Center at San Antonio, San Antonio, TX 78229, USA; farokhi@uthscsa.edu

**Keywords:** refugees, immigrants, quality of life, children’s oral health, DMFT/dmft, dental caries

## Abstract

*Objective:* The study assessed a preventive outreach educational intervention targeting improvements in dental caries and oral-health-related quality of life in the children of refugee families by comparing pre- and postintervention outcomes. *Methods:* This randomized controlled clinical trial assessed the outcomes at baseline and three times over six months using the WHO oral health assessment form (DMFT/dmft) and the parent version of the Michigan Oral-Health-Related Quality of Life scale. Children and at least one of their parents/caretakers were educated on oral health topics in two one-hour sessions. *Results:* Of the 66 enrolled families, 52 (72%) completed the six-month follow-up. DMFT/dmft scores increased significantly in both the control and intervention groups (*p* < 0.05); differences in the changes in the DMFT/dmft and MOHRQoL-P scores from baseline to the three- and six-month follow-up visits between groups were not significant (*p* > 0.05). *Conclusions:* Oral health education programs targeting a diverse group of refugee children and their parents/caregivers single-handedly did not reduce the increased number of caries lesions or improve oral-health-related quality of life.

## 1. Introduction

Children and adolescents living in poverty and underserved areas suffer more from dental caries than their peers, and their dental disease is more likely to be left untreated [1]. Dental caries can cause severe pain, soft tissue infections, abscesses, difficulty in chewing, malnutrition, gastrointestinal disorders, poor speech articulation, and low self-esteem [2]. Dental caries negatively affect the learning potential and academic performance of some children because the pain interferes with their ability to concentrate and, in severe cases, with their nutrition [3].

Over the last two decades, the prevalence of dental decay has decreased significantly in the U.S. population [4]. This decrease is mainly attributed to the success of preventive practices, such as the fluoridation of water, improved oral hygiene, and the application of sealants aimed at protecting teeth from decay [5]. However, this improvement is not reflected in the oral health of underserved communities, including low-income families, refugees, and immigrants.

Caries are particularly prevalent in children from low-income families who are unable to afford dental care [6]. Consistent with the overall health disparities in the U.S., families from racial or ethnic minority groups and families with lower socioeconomic status have the greatest prevalence of oral diseases [7]. For instance, children of migrants workers have the highest prevalence of dental diseases among schoolchildren in the U.S [8].

Poor oral health amongst refugees is related to limited access to dental care in the refugee camps [9]. Traditionally, refugee children have limited access to dental care for many years prior to resettlement, may have not been exposed to oral hygiene instructions, and may suffer from fractured teeth, tooth decay, and abscesses. Upon arriving in host countries, dental care may be expensive and out of reach [10]. Dental care is also affected by cultural practices and beliefs, such as fear related to tooth extraction and the routine extraction of anterior teeth or practices of brushing with a stick rather than a toothbrush [10,11].

Despite the evidence that treating oral diseases or preventing dental caries among children will improve children’s quality of life [12,13], currently, studies have not assessed the effects of preventive outreach educational approaches to improving oral health and its related quality of life in underserved refugee communities in the U.S. This study hypothesized that a preventive oral health educational program administered to parents of refugee children would reduce their incidence of dental caries and subsequently improve children’s oral-health-related quality of life. The aim of this study was to evaluate the preventive outreach oral-health-related quality of life and dental caries experience after the administration of an educational intervention program targeting refugee children.

## 2. Materials and Methods

### 2.1. Study Design

The design of this randomized controlled clinical trial followed the guidelines published by the Consolidated Standards of Reporting Trials (CONSORT) [14]. The Institutional Review Board approved this study of the University of Texas Health Science Center at San Antonio (HSC20170703N). Before enrolment, every child’s parent/guardian received and signed an informed consent form. This study is a part of an educational intervention project that was conducted for refugee children and their families; the cognitive measures of the educational components of this project were previously tested and published; therefore, this study is a continuation of the previously published paper with similar a methodology and different measurement outcomes [15].

### 2.2. Study Population

The study’s target population was parents or caregivers in refugee communities and their children residing in San Antonio at Bexar County in Texas, U.S.A. The study recruited a parent/caregiver from a family with at least one child under 12 years old through the local Catholic Charities and the Center for Refugee Services. The study population members spoke one of the following primary languages: English, Nepalese, Turkish, Spanish, Burmese, and Arabic. The study targeted recent refugees from a more culturally diverse group of countries: Eretria, Cameroon, Myanmar, Turkey, Nepal, Iraq, and Afghanistan.

The study needed thirty-seven participants for the clinical oral health assessment and 55 participants to evaluate oral-health-related quality of life to detect an effect size of a 20% difference between the two groups with a power of 80% and a *p*-value level set of 0.05. The study recruited more than 60 participants to account for potential dropout during the study.

### 2.3. Inclusion and Exclusion Criteria

Inclusion criteria included being a recent refugee and a caregiver of at least one child under the age of 12 years residing with nonrefugee families, staying in San Antonio, and not planning to move outside the city during the six-month study period. The exclusion criteria were being part of a refugee family that spent more than one year in the U.S. and a history of current dental care.

### 2.4. Study Procedures

The baseline survey was distributed to all participants who consented upon enrolment to the project, i.e., Time 1 (T1), and immediately after the educational session, i.e., Time 2 (T2), which focused on one caregiver and one child per family. The study conducted two follow-up visits to evaluate the intervention’s effectiveness three and six months after the educational session. Multilingual, trained research assistants administered all surveys with assistance from interpreters in the six above primary languages.

Upon enrolment, study individuals were randomly divided into intervention and control groups: participants in the first group agreed to attend five educational classes and four assessments, and participants in the second group consented to complete 2 study assessments without the educational intervention. The randomization process used a computer-generated list of random numbers to allocate the participants. The study evaluated the balance between the control and intervention arms by comparing the demographic characteristics and outcomes measurement at baseline using two-sample *t*-tests. While participants and educators were aware of each child’s allocation, the study coordinator blinded the outcome evaluators and data analysts to the allocation.

### 2.5. Educational Intervention

An appropriate, culturally centered educational intervention was administered after enrolment using two different educational models. The first is “A Healthy Mouth for Your Baby,” which is an educational pamphlet for parents that discusses the importance of oral hygiene in children. The pamphlet was developed by the U.S. Department of Health and Human Services under the National Institute of Dental and Craniofacial Research, Washington, USA. The topics include the importance of primary teeth and the role of oral hygiene and fluoride in preventing dental caries, feeding, and diet, and the value of a dental visit by the age of 1 year [16]. This recourse is validated by the Nutrition Education Subcommittee in the NIH and the Nutrition Policy Board Committee on Dietary Guidance in the DHHS and the Dietary Guidance Working Group in the U.S. Department of Agriculture in Washington, USA [17].

The second educational guide utilized was the “Healthy Habits for Happy Smiles”, which comprises a set of brochures to encourage good oral health habits for pregnant mothers and parents of toddlers, infants, and children. The office of Head Start produced the resources under Cooperative Agreement No. 9OHC0013 by the National Center on Early Childhood Health and Wellness, Administration for Children and Families, DHSS [18]. All of the educational materials utilized during the intervention were translated to the different native languages of the refugee families who participated in the project. These guides were tailored for the refugee participants in the program and edited to be appropriate culturally and linguistically.

This educational program included two one-hour classes of illustrations and guidance using visual materials highlighting the following topics: fluoride application, oral hygiene, nutrition, oral health, and dental care access, including preventive measures. Demonstrations and instructions were performed by research volunteers and interpreters from different cultural communities, which reflected the multifariousness of the refugee families. Emphasis was placed to deliver the educational interventions with assistance from volunteer interpreters within the same background as each refugee participant to improve their effectiveness and motivational interviewing skills. Investigator dentists calibrated research assistants with interpreters and the refugee community members. A primary focus was to implement the culturally and linguistically appropriate delivery of this educational approach. The research assistants distributed educational brochures in the participants’ native languages and children’s toothbrushes and toothpaste tubes at the end of each class and visit. Following the protocol, the control participants received the educational intervention classes at the end of the study.

### 2.6. Outcome Measurements

#### 2.6.1. Michigan Oral-Health-Related Quality of Life Scale–Parent Version (MOHRQoL-P)

The children’s oral-health-related quality of life was measured with the MOHRQoL-P scale [19]. This scale was originally developed as a multidimensional measure of children’s oral-health-related quality of life from the parent’s perspective. It can be used for children who are too young to answer the questions or who have special healthcare needs that do not allow them to respond to items directly. Responses are provided on a 5-point rating scale ranging from 1 = “strongly disagree” to 5 = “strongly agree.”

#### 2.6.2. WHO Oral Health Assessment Form

Children were clinically assessed according to the WHO oral health assessment form, which counts the sum of decayed, missing, and filled teeth (DMFT for permanent teeth and dmft for primary teeth) [20]. This measure was performed at T1 and T4 by two dentists to quantify the changes in DMFT/dmft between these time points. Dental examinations were completed through a clinical assessment based on the WHO oral health assessment form. Radiographs and dental history were not obtained. The level of caries in the primary and permanent dentition based on the DMFT/dmft score for children ≤12 years of age was classified according to the WHO oral health assessment as follows: very low <1.2, low 1.2–2.6, moderate 2.7–4.4, high 4.5–6.5, and very high >6.5.

### 2.7. Statistical Analysis

Statistical analyses were performed using SPSS statistical package version 23 (SPSS Inc., Chicago, IL, USA). The generalized estimating equation (GEE) was used to test the hypothesis that the educational intervention would positively affect the oral-health-related quality of life and DMFT/dmft of children [21]. The GEE multivariate regression model analyzes the prospective data and estimates the regression parameters that account for within-subject correlations of dependent variables’ responses.

Each regression model was analyzed according to the better-fit working correlation matrix. The biostatistician performed model selection to distribute the residual error for all outcomes and choose the distributions that showed the lowest quasilikelihood information criterion (QIC) scores. An exchangeable correlation matrix model was selected as the most appropriate one. Socioeconomic (monthly household income) and education status were asked because we also hypothesized that participants with better income and education levels would show more remarkable progress. The survey’s reliability evaluated using Cronbach’s alpha after averaging and summarizing the MOHRQoL-P scores. Intraclass correlation coefficients (ICCs) and their 95% confidence intervals were calculated based on single-measurement, absolute agreement, and 2-way mixed-effects models to examine intraexaminer reliability.

## 3. Results

### 3.1. Demographics of the Participants

The majority (74%) of the parent/caregiver participants were female, 96% were married or living with partners, 51% had completed high school education, and 71% of the participants had a monthly income of less than $2000. The individuals’ demographic characteristics at baseline are presented in Table 1.

**Figure 1:**
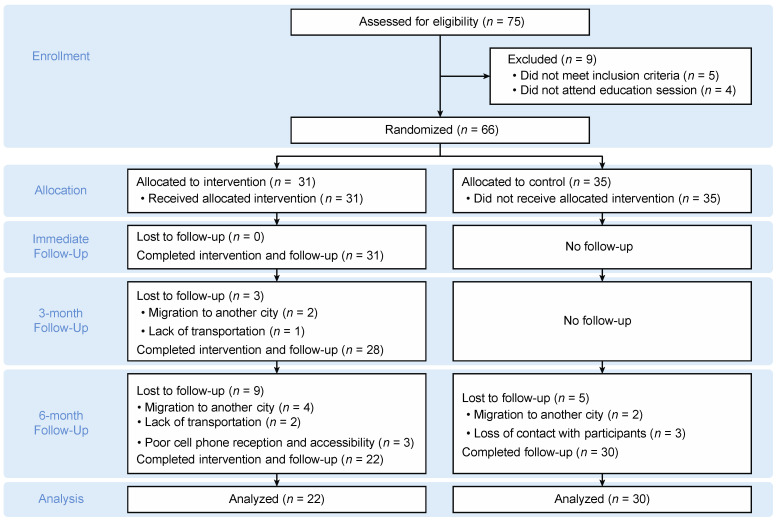
Flow diagram of the recruitment of the participants in the intervention group and the control group.

Eligible participants were recruited from January 2018 to August 2018. Participants attended study visits at the time of randomization (baseline) and three times over six months. The dropout rate was 28% at the six-month (T4) follow-up. Potential reasons for this dropout rate were recorded as lack of transportation, poor cell phone reception, the inability to contact participants, and migration to another city for work. The study groups’ distribution and sample sizes across the four measurement time points are displayed in Figure 1.

### 3.2. Verification of Randomization and Intraexaminer Reliability

The outcomes before the intervention at T1 between the intervention and control groups were compared by using two-sample *t*-tests. All *p*-values were >0.6; therefore, we concluded that the randomization was successful. The ICC for intraexaminer reliability was 0.82, with a 95% CI of 0.79–0.85, which indicates good reliability.

### 3.3. Outcomes

DMFT/dmft Score

**Figure 2:**
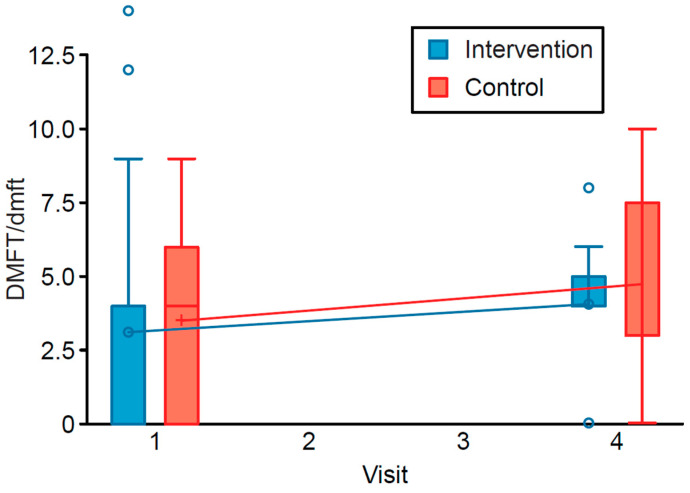
Distributions of DMFT/dmft scores in the intervention and control groups before and after the administration of the intervention program.

The DMFT/dmft score for the entire study population (e.g., the pooled intervention and control groups) significantly increased at six months after the intervention (T4) compared to the preintervention baseline score (T1). The regression coefficient β estimate (and its 95% CI) for the intervention was 0.2814 (0.0605, 0.5023), with *p* = 0.0125. After adjusting for income and education levels, the respondents who had received the educational program did not differ significantly from the participants who did not receive it in terms of the DMFT/dmft score (intervention β = −0.2310, 95% CI (−0.5733, 0.1113), *p* = 0.1859). Detailed descriptions of the results of the multivariate regression analyses are shown in Table 2. The distribution of DMFT/dmft by intervention and control status at each time point is shown in Figure 2.

### 3.4. MOHRQoL-P Score

Given the multidimensional nature of the MOHRQoL-P scale, indices of parents’ perceptions of their children’s oral-health-related quality of life were constructed. The items were averaged to create two indices: “interference” and “function.”

The “interference” index was interpreted and averaged as the parent’s perception of how much their child’s oral health status interfered with the child’s life. The reliability of this index was indicated by a Cronbach’s alpha value of 0.80. The “function” index was interpreted as the parent’s perception of how their child’s oral functioning affected the child’s life. The reliability of this index was indicated by a Cronbach’s alpha value of 0.78.

GEE was performed to test whether the preintervention (T1) and postintervention (T4) “interference” and “function” index values differed significantly. For the entire study population (e.g., the pooled intervention and control groups), significant differences were not observed between the children’s pre- and postintervention oral-health-related quality of life (interference: β = −0.0223, 95% CI (−0.0810, 0.0364), *p* = 0.4562; function: β = −0.0166, 95% CI (−0.0915, 0.0583), *p* = 0.6638) after adjusting for socioeconomic status and education level. Detailed descriptions of the results of multivariate regression analyses are shown in Table 3.

## 4. Discussion

The main finding of this study is that the administration of an oral health education program to the parents of the children from refugee families did not improve their oral-health-related quality of life or reduce dental caries compared to control participants who did not receive the intervention. The DMFT/dmft score increased significantly in the entire study population (i.e., the pooled intervention and control groups) at six months after the intervention (T4) compared to the preintervention baseline DMFT/dmft score (T1), and the differences between the intervention and control groups were not statistically significant. This increase in the DMFT/dmft score might be due to either an increase in dental carious lesions or previously treated cavities, as filled and unfilled cavities contribute equally to the DMFT/dmft index.

These findings are inconsistent with the results of studies of the effects of oral health practices on children in the general U.S. population [22,23,24]. A possible reason for this difference is that this intervention was mainly focused on an educational program aiming at upstream preventive approaches to reduce oral health disparities rather than oral health promotion in general. Studies have investigated interventions involving dental treatment, rehabilitation, and oral health promotion to significantly improve quality of life. For instance, Low, Tan, and Schwartz [22] studied the effect of severe caries on the quality of life of young children and concluded that preschool children with dental caries did not necessarily complain of pain but instead manifested the effects of pain as changes in their eating and sleep habits. Moreover, Acs, Pretzer, Foley, and Ng [23] studied perceived outcomes and parental satisfaction following dental rehabilitation under general anesthesia and found that parents perceived an improvement in the quality of life of their children following comprehensive dental rehabilitation, with the most considerable improvement noted in pain experience, followed by an improved ability to eat and sleep. Similarly, Thomas and Primosch reported a significant improvement in the quality of life of young children with severe caries who received complete dental rehabilitation under general anesthesia, as reported by their parents [24].

Additionally, nonmodifiable (socioeconomic background) and modifiable (oral-health-related knowledge and habits) risk factors for refugee children from families with a higher socioeconomic status, and parental education levels did not yield better oral-health-related quality of life. In addition, parents’ assessments of their child’s quality of life after the completion of the educational program and during the six-month follow-up period showed nonsignificant decreases. A possible explanation for this decrease is that families became more aware of oral-health-related quality of life issues than they were at the initial preintervention stage.

Furthermore, studies have linked a decrease in refugees’ oral health to less utilization of preventive oral hygiene and less access to preventive care [25]. Refugees become more susceptible to poor oral health upon adopting a Western diet, particularly if they do not have access to dental care or have not adopted acceptable oral hygiene practices [26].

The main methods we used in our work health education program were instruction, demonstration, and motivational interviewing. Although we used a motivational interviewing technique, our study did not successfully change the outcomes for oral-health-related quality of life. However, one review noted that motivational interviewing had conflicting results for improving oral health behavior since it yields a positive impact for studies that were conducted in a clinical setting and applied interventions in an individually tailored manner. In contrast, it did not produce a positive result for studies conducted at the population level [27]. Our study’s results are in agreement with the conclusions of the review by Cascaes et al. since the motivational interviewing technique in our trial was not individually tailored to each participant and was not performed in a clinical setting.

Longitudinal interventions, such as preventive outreach, restorative, and curative dental treatments, are needed in addition to education programs further to improve dental caries experience and oral-health-related quality of life. Another possibility for the limited oral health improvement of the newly arrived refugee families is their immediate oral healthcare barriers, which include health, financial, and information literacies. During the study’s motivational interviewing stage, the participants expressed that their initial priorities were to acquire skills to gain employment, learn English, and secure housing and educational opportunities, which may take precedence over accessing oral health care services. In contrast, for the U.S. mainstream population, access to preventive and restorative dental services plays an essential role in their oral health status [28].

Understanding that many refugees come from camps where upstream preventive oral health services are not a priority is another reason for their barriers to oral health care. They mainly function in the downstream aspects of accessing oral health as a need basis-only approach.

The study has several limitations. First, it had a low power to detect the effects of the trial because of the small sample size. Second, the possible inaccurate dental caries diagnosis based on a clinical examination without radiographic assessment. Third, the DMFT/dmft index may not be the best outcome measure as it assesses the teeth but not the gingiva, which may be more rapidly responsive to improved oral hygiene, and other dental caries measures would be more accurate like the Pulp Ulceration Fistula Abscess index (PUFA) and International Caries Detection and Assessment System (ICDAS).

## 5. Conclusions

Our findings for public health imply that oral health education alone is not sufficient to improve dental caries risk or oral-health-related quality of life in underserved refugee children. Clinical preventive and curative measures may also play an important role in improving caries prevalence, particularly among lower oral health literacy. Studies evaluating more comprehensive intervention programs with more extended follow-up periods and more comprehensive dental outcomes that are preventive oriented are needed. Recent refugees need to be more informed about the relationship between oral and general health and the importance of regular preventive dental checkups even when they have no pain or acute complaints to reduce the gap between their existing knowledge and practice. It is important for oral health promotion campaigns to bridge the gap between knowledge and practice and concentrate more on preventive oral health practice and oral health literacy.

## Figures and Tables

**Table 1:** Demographic characteristics of the parents/caregivers.

Variables	Intervention Group	Control Group	Total
	% (*n*)	% (*n*)	% (*n*)
Gender			
Male	23 (7)	37 (13)	30 (20)
Female	77 (24)	63 (22)	70 (46)
Marital status			
Married/living with partner	94 (29)	100 (35)	97 (64)
Single/separated/divorced/widowed	6 (2)	0 (0)	3 (2)
Education			
Elementary or middle school	52 (16)	49 (17)	50 (33)
High school	19 (6)	17 (6)	18 (12)
Some college but not a degree	19 (6)	6 (2)	12 (8)
Bachelor’s degree	7 (2)	17 (6)	12 (8)
Master’s degree	3 (1)	11 (4)	8 (5)
Race			
Southeast and South Asian	58 (18)	60 (21)	59 (39)
Middle Eastern	32 (10)	20 (7)	26 (17)
African/Hispanic	10 (3)	20 (7)	15 (10)
Country of origin			
Myanmar	49 (15)	31 (11)	39 (26)
Nepal	26 (8)	14 (5)	20 (13)
Turkey	13 (4)	20 (7)	17 (11)
Iraq	3 (1)	20 (7)	12 (8)
Afghanistan	3 (1)	6 (2)	4 (3)
Cameroon	3 (1)	3 (1)	3 (2)
Eritrea	3 (1)	3 (1)	3 (2)
Mexico	0 (0)	3 (1)	2 (1)
Language			
Burmese	48 (15)	31.4 (11)	39 (26)
Nepalese	26 (8)	14.3 (5)	20 (13)
Turkish	13 (4)	20 (7)	17 (11)
Arabic	3 (1)	20 (7)	12 (8)
English	10 (3)	12 (4)	10 (7)
Spanish	0	3 (1)	2 (1)
Monthly income			
<$2000	64 (20)	80 (28)	73 (48)
>$2000	36 (11)	20 (7)	27 (18)
Parent or caregiver’s Age			
18–24 years	16 (5)	17 (6)	17 (11)
25–34 years	45 (14)	49 (17)	47 (31)
35–44 years	29 (9)	31 (11)	30 (20)
>45 years	10 (3)	3 (1)	6 (4)
Child’s age			
3–5 years	29 (9)	28 (10)	29 (19)
6–8 years	45 (14)	43 (15)	44 (29)
9–11 years	23 (7)	26 (9)	24 (16)
12 years	3 (1)	3 (1)	3 (2)

**Table 2:** Multivariate analyses of the predictors of the DMFT/dmft outcome.

Outcome	Parameter	Estimate	95% Confidence Interval		*p*-Value
DMFT/dmft	Intervention	−0.2310	−0.57	0.11	0.18
	Time (T4 vs. T1)	0.2814	0.06	0.50	0.01
	Income (high vs. low)	−0.1293	−0.60	0.34	0.59
	Education	−0.0773	−0.20	0.05	0.24

**Table 3:** Multivariate analyses of the predictors of oral-health-related quality of life outcomes.

Outcome	Parameter	Estimate	95% Confidence Interval		*p*-Value
Interference	Intervention	0.02	−0.04	0.10	0.45
	Time (T4 vs. T1)	−0.02	−0.08	0.03	0.45
	Income (high vs. low)	−0.03	−0.14	0.08	0.60
	Education	0.01	−0.02	0.04	0.50
Function	Intervention	−0.04	−0.16	0.08	0.52
	Time (T4 vs. T1)	−0.01	−0.09	0.05	0.66
	Income (high vs. low)	−0.06	−0.25	0.12	0.47
	Education	0.02	−0.03	0.08	0.39

## Data Availability

Not available for publicity.
